# Editorials

**Published:** 1910-04

**Authors:** 


### EDITORIALS

The Business Office of the JOURNAL-RECORD is 1 1-2 to 5 1-2 South Broad Street.
The Editorial Office is 1014-15 Century Building.
Address all Business Communications to *Journal-Record of Medicine*, 1 1-2 to 5 1-2 South Broad Street.
Make remittances payable to *THE JOURNAL-RECORD OF MEDICINE.*
On matters pertaining to the Editorial and Original Communications, address Edgar G. Ballenger, M. D., Atlanta, Ga.
Reprints of Original Articles will be furnished at cost price. Requests for the same should always be made in THE MANUSCRIPT.
We will present, postpaid, on request, to each contributor of an original article, twenty (20) marked copies of THE JOURNAL-RECORD OF MEDICINE containing such article.

### THE POSTAL DEFICIT.

The postal deficit of seventeen million dollars has lead the postmaster-general of the United States to make the remarkable and short-sighted statement that this shortage is due to second class matter. For good and apparently sufficient educational reasons, years ago, it was deemed expedient to allow a rate of one cent per pound for second class matter. This has undoubtedly been one of the causes for the enormous number of magazines and journals published in this country with their enormous educational value. Aside from this factor in education, one must also consider the business fostered or actually created by the advertising pages of second class matter and of the enormous amount of first class matter directly the outcome of this advertising.

So apparent is the good derived by the country at large from this source, it seems astonishing that, with the much larger expenditures upon the army and navy with less apparent advantages, an increase in the rate on second class matter should be contemplated with the necessary curtailment of its advantages.

Under similar circumstances, a business firm would first of all investigate its contract with the railroads, (this phase of the situation, however, does not reflect much credit upon the department) ; or it would consider the matter of increasing the revenue from the least profitable part of the business, which is the rural free delivery; the establishment of the much needed parcel post would not only make of it a great convenience, but would also bring sufficient new business to make up the present deficiency.

Those familiar with the subject admit that the railroads are both overpaid and underworked. This being the case, why should not the increase of business brought by the parcel post be sought? Why should express companies obtain much greater reductions in rates than the postal service?

There is no necessity for the government to pay special rates for unusually rapid transportation of second class matter. Why not ordinary transportation at more reasonable rates? The government pays $1,000 a year for each mail car and $50,000,000 for transportation. "If it would seek instead of declining business," it would probably get out of the railroads double the service it now gets, and soon would have a surplus instead of a deficit.

### PUBLIC PROTECTION FOR CHARLATANS.

Assemblyman Grinnell has introduced into the Ohio State Legislature a bill to prohibit the advertisement of certain diseases which are so constantly flaunted in the daily and other papers (especially here in Atlanta). It would give us great pleasure to see a similar bill passed by the Georgia Legislature, but such a thing is not easy to obtain for the simple reason that the papers which are such important factors in forming public sentiment and crystallizing it into laws derive so much profit from these vicious advertisements that their support cannot be relied upon. If, however, an influential legislature can be induced to introduce such a bill and if the physicians of this State will trouble themselves "one small bit" to urge their representative to support the bill, the matter would not be an impossibility by any means. I fear, however, our minds are too deeply anaesthetized by custom and tolerance of these evils to even hope that we will awaken from our lethargy.

The text of the bill is as follows: "Any person, firm, association, or corporation who shall print, publish, or circulate any advertisement of or concerning any means, device, or method for the treatment, cure, alleviation, or prevention of any venereal, sexual, or menstrual disease, weakness, or condition, shall be fined not less than twenty-five dollars nor more than three hundred dollars; providing that nothing herein shall prevent such printing, publication, or circulation in exclusively medical publications not designed primarily for public inspection, or in trade journals circulated among dealers and not designed primarily for public inspection."

---

### A REMARKABLE INSTITUTION AT WHOSE HEAD IS A REMARKABLE MAN.

The institute referred to in the title is the American Woman's League and the man is Edward Gardner Lewis, who may truly be called a genius. A genius is one who conceives an unusual or wonderful idea and develops it with infinite pains. Mr. Lewis is such a man and the American Woman's League is worthy of his effort.

The purpose and management of this institution is so unusual that we wish to mention some of the salient features, hoping that in this manner we may arouse the women in the South to avail themselves of its valuable opportunities, which may be obtained without the expenditure of money and with little individual effort.

Courses of instruction may be obtained in the following branches by mail or personally:

| | |
|---|---|
| DRAWING | PENMANSHIP |
| PAINTING | KINDERGARTEN COURSES |
| SCULPTURE | TYPEWRITING |
| JOURNALISM | STENOGRAPHY |
| PHOTOGRAPHY | BOOKKEEPING |
| AGRICULTURE | COMMERCIAL LAW |
| HOME ECONOMICS | HISTORY |
| CIVIL SERVICE COURSES | CHEMISTRY |
| COURSES FOR TEACHERS | LITERATURE |

The Woman's League is already one of the most powerful organization of women, and its benefits are being given daily to thousands of persons throughout the United States. Membership in the League entitles one, for life, to the benefits of the university. Where sufficient members are present, towns have local chapters which afford many social and intellectual advantages. Investigations of University City, the home of the Woman's League, have revealed most wonderful plans and development of this great business and educational structure. Hearty co-operation on the part of the women of America will ensure for themselves extraordinary advantages. Being a very desirable resident suburb of St. Louis, the real estate in University City and owned by Mr. Lewis is now worth more than a million dollars. Success must follow Mr. Lewis' plan if the women of America realize the great opportunities now within their reach.

---

### CHILDREN'S TEETH.

Philanthropists seem to be vieing with each other in making unique and useful donations; one of the most sensible of such donations was recently made by Thomas A. Forsyth of Boston, who has given two million dollars to be used as a perpetual fund whereby children from their birth to their sixteenth year may receive free and skilled treatment for their teeth. At first glance this might appear to be a freaky gift and yet it is doubtful if any philanthropy of recent times will be productive of greater good. Dental defects are the commonest affection of school children, and are due almost entirely to neglect. Decaying teeth are known to be the cause, or act as a contributing cause of many more or less serious ailments. From the physical as well as sociologic standpoint it is of greater importance for boys or girls to have good teeth than it is for them to know algebra and such studies.

